# The Impacted Lower Third Molar

**Published:** 1921-05

**Authors:** C. Edmund Kells


### THE IMPACTED LOWER THIRD MOLAR

BY C. EDMUND KELLS, D.D.S.

Judging from a number of comparatively recent programs of dental societies, the impacted lower third molar still "holds its place in the sun."

For many years the removal of impacted teeth has been a matter of intense personal interest, and we have continually studied these cases, endeavoring always to simplify the operation for their removal and minimize the pain so often following the operation.

Former papers have described an operation devised by the writer for the removal of these teeth, and year by year has seen it modified more or less, always with beneficial results; and during the past two years such radical changes have been made, which have so *steadied* the post-operative stage, or rather practically eliminated the post-operative stage, as to appear to warrant a report upon them.

The operation for the removal of these deeply embedded lower third molars, as usually performed, may well be considered as consisting of two stages: the operative stage, usually a painless one; and the other, the post-operative stage, usually one of suffering and discomfort. And it is undoubtedly upon the character of the treatment which the patient receives during the former stage that the second stage depends.

For the surgical removal of impacted lower third molars, the following postulates are offered:

1. All trauma possible should be avoided.

2. The soft parts should be lacerated to the least possible degree.

3. Only that amount of bone should be cut away, and no more, as will allow the extrusion of the tooth.

4. Both surgical and mechanical shock should be eliminated as far as possible.

5. None but the best and purest of solutions should be injected.

6. The operative field should first be most thoroughly sterilized.

7. Surgical asepsis should be maintained throughout the operation as fully as circumstances will permit.

It is believed that the operation as now to be described fully meets the above postulates, and it is also now believed that *whenever* post-operative pain has occurred in the past *in our own practice,* it has been due to one or more of the following reasons:

1. First and foremost, it is believed that the heat caused by cutting through the dense enamel and dentin, or even bone, has been the prime cause of the resultant suffering.

2. Ragged edges of the socket may have been left to irritate the overlying tissues.

3. Splintering of the margins of the socket may have occurred, causing small parts to exfoliate later on.

4. Possibly the soft tissues were unduly lacerated.

With these conditions in mind, every effort is now made to eliminate each of those faults, and when they are successfully eliminated, there is absolutely no post-operative stage.

### THE REFRIGERATING PLANT

In order to eliminate the heat produced by the bur or cutting stone, all such cutting is now done under a stream of iced distilled water. At first iced saline solution was used, but one day a patient was nauseated by the salt water and nearly spoiled a perfectly good operation, so distilled water has been used ever since.

The apparatus used for this purpose consists of a flask for holding freshly distilled water, a jar for holding cracked ice and water, and a cooling tube to be immersed in same. Thus the distilled water does not come in direct contact with the ice and, consequently, is not contaminated.

The cooling tube is connected to the handpiece or

"duplex tip" by rubber tubing, of course, and suitable devices are provided for controlling the flow of this iced distilled water.

An aspirating machine is required to remove the iced water from the mouth, as its flow is too great for the capacity of the saliva tube; therefore, an electrically operated vacuum pump with its foot control and the receiving jar of any kind with its proper rubber tubing connections are necessary to complete this part of the installation.

### PREPARING THE PATIENT

A five-grain bromural tablet is usually given beforehand to all nervous patients (this means to about all ladies), with instructions to take it about one-half hour before appointment.

### THE SKIAGRAPH

A perfectly satisfactory skiagraph is absolutely necessary, which must be studied carefully, for upon the shape and number of the roots of the tooth depends in a measure the choice of procedure, as far as the details of the operation are concerned.

The film is secured in a suitable holder and placed upon the bracket table—always in plain sight during the operation.

### THE NOVOCAIN SOLUTION

A minimum amount of suprarenin is used in the novocain solution, under the impression that the less disturbance to the tissues by the use of adrenalin the more rapidly will they recuperate. There is no necessity for *our* reducing the hemorrhage, because all blood is aspirated and not a *single sponge is used during the entire operation.* As a matter of fact, it is believed that some hemorrhage is preferable to an absolutely bloodless field.

The greatest care is given to the freshly distilled water and solution, needles, syringes, etc., and equal care is taken to insure the dryness of the mucous membrane before sterilizing with tincture of iodin, and to insert the needle in a *dry* field.

An ordinary eight-ounce De Vilbiss bottle can be had with fittings which throw a stream instead of a spray. The nozzle which came with the bottle was removed, and a suitable "tip" was connected to the bottle by about twelve or fifteen inches of very flexible rubber tubing. This allows of the delicate manipulation of the tip which is impossible with the bottle and tip as sent out by the factory.

The bottle, which is charged with Dakin's solution, is held by an assistant who manipulates the valve upon signal, while the operator holds the tip (made by the writer for this special purpose), which he runs all around the mouth, following the festoons of the gums both lingually and bucally. Thus the force of the stream frees the interdental spaces as well as the surfaces of the teeth of all extraneous particles and mucous. Then all the mucous membranes of the mouth are flushed off thoroughly, thus cleaning the entire mouth most satisfactorily. We thus have a pretty clean mouth to work in, as no spray will accomplish what this stream will do. From three to five pounds of air pressure are used, and the saliva tube carries off the solution.

### ASSISTANTS

The method of operating as now being detailed, really requires the services of two well-trained assistants in order to *get the most out of it.* The one stands on the left side of the chair, operating the aspirating and refrigerating machines, assists with a retractor when necessary and also in many other ways. The other stands on the right and looks after the operating instruments, engine, etc., and saves the operator much time. However, one can get along with only one assistant, who then must stand on the left side of the chair and operate the machines.

### THE PATIENT'S COMFORT

It is absolutely essential that the patient be placed in a perfectly comfortable position and maintained so during the operation. As it is not unusual for the operation to be completed without the patient once closing the mouth. the use of a mouth prop is frequently advisable.

### MOUTH PROP

Many years ago the writer designed one for his own use. Its principal and original feature is that it does not hold the mouth in a *fixed position.* The patient can move the lower jaw to a certain extent which gives relief, and yet it stays in place all the time without having the mouth propped open to the widest degree possible. Another feature—its adjustability while in the mouth—is also most valuable. It is simplicity itself and was made with a spring which keeps it in place and is held by a pad of chilled modeling compound for stability.

### AN IMPORTANT STEP

The next step—that is uncovering the surgical field—is a most important one. While we must bear in mind that the less we lacerate the tissues, the less the trauma, still a greater evil is not obtaining a sufficiently large field, in which case the soft tissues will be lacerated unnecessarily because they are in the way. Again, we must see what we are doing in order to do it well. As a matter of fact, cutting a sufficiently long flap and holding it well out of the way so that it will not be injured during the operation, causes less laceration in the end. The character of the flap is also most important.

### CHOICE OF INCISIONS

To run the knife (and it should be a sharp one) down the center of the field would naturally result in two flaps to handle, one on each side of the cut. To make the incision along the buccal edge of the operative field would give only one flap it is true, but *we* find it practically impossible to take care of such a flap with ease and satisfaction. Therefore, the incision is made *along the lingual border* of the field to be exposed, and thus there is only one flap which is to be retracted bucally—the most rational way to cut and handle a flap, it would appear.

### THE ASPIRATING MACHINE

From the moment the knife penetrates the soft tissues, the assistant keeps the operating field in plain view and free from blood by means of the aspirating machine. Thus being able to see what one is doing all the time, and not having to stop to sponge, is a factor of inestimable value, and undoubtedly in some instances cuts down the operative time at least fifty per cent., an item of the greatest interest considered from any angle whatsoever. Besides this advantage, the eliminating of all sponging is of the greatest benefit, because sponging does cause trauma and trauma, you know, must be avoided. The operator who does not use an aspirating machine deprives both himself and patients of one of the greatest aids to a satisfactory operation.

### THE RETRACTOR

From time to time attempts have been made to design a satisfactory retractor, as none has ever been found on the market; but so far no very marked success has been achieved. Recently it occurred to us that the very idea of a retractor for the flap in question was wrong, so an entirely different style of instrument was conceived. Retractors, as a rule, must have teeth in order to tear into and grip the periosteum—the very idea of which is objectionable.

### FLAP HOLDER

Now we are working along an entirely different line. Instead of *retracting* the tender flap by gripping it and thus injuring it, we first push it away without injury, and then we plant our flap holder firmly against the bone and thus the flap is held entirely out of the way without any tension upon the periosteum or other soft tissues. This appears to be a great improvement, although the one now used is a rather crude affair, subject to alterations any day.

### CUTTING THE FLAP

Before making the cut, the parts are thoroughly dried and well swabbed with iodin. The incision starts from the disto-lingual angle of the second molar and runs as far back as is considered necessary, and then bucally for the required distance. The gum is then well separated from the distal margin of the second molar, and also from the distal half of its buccal surface. From this point the knife is run straight down (bucally of course) for a distance of a half inch more or less as may be required. The gum is also separated from the distal half of the second molar upon its lingual side.

Originally our own flap did not extend along the buccal wall of the molar. This idea was gotten from Dr. Charles M. Proctor of Boston, and is a *very great* improvement on the old one, as it gives a much better view of the operative field.

With the flap thus held out of the way by the operator himself with his left hand, an ample working field is exposed to view by means of the aspirating machine, and then each tooth becomes a problem unto itself, but the general principles hold good.

### EXPOSING THE TOOTH

When the tooth is entirely covered with bone, chisels are used to remove only that bone which is superimposed upon the crown of the tooth.

### CUTTING THE TOOTH IN TWO

Having thus exposed the crown, or rather the distal surface (top), to view a three-eighths-inch knife-edge carborundum stone in a straight handpiece is used to cut a nick in the dense enamel, so that an engine drill will take hold. The deeper one can cut this nick without encroaching upon the bony plate upon either side of the molar, the better it is; *but one must not cut these bony plates with this stone.* A little judgment is necessary in deciding just where this nick is to be started, or, in other words, how much of the crown is to be cut off, for there is such a thing as cutting off too little, and it is just as possible to cut off too much. A study of the skiagraph is essential in order to decide where and at *what angle* it is best to cut each tooth.

A spear drill is next used in the contra-angle handpiece by which a hole is drilled right down through the crown of the tooth and *no further*—if possible. The next step is to me the most difficult one in the whole operation, and one upon which the post-operative stage, if there is to be a post-operative stage, very largely depends, and that is the cutting of the tooth in two without injury to the lingual or buccal wall or soft tissues.

The cutting is done by means of special burs in the contra-angle handpiece, using a perpendicular sawing motion and turning the handpiece from side to side. They are extra long cross-cut fissure burs.

If the lingual plate is encroached upon to any appreciable extent by the bur, trouble is sure to follow. If the bur goes down beyond the tooth to any great extent, the inferior dental nerve may possibly be injured, in which event the anesthesia (?) may last for several months. This has happened to the writer once only. Unnecessary cutting of the buccal plate is less apt to cause trouble, but, of course, should be avoided if possible.

As just stated, this is a very difficult stage in the operation, and in order to perform it properly, one should have the operative field in plain view by a thorough retraction of the soft tissues and the use of the suction machine. While cutting in the direction of the lingual plate, the flap holder should be pushed down between the plate and the gum and held there in order to protect the soft tissues if necessary.

### REFRIGERATING THE PARTS

At the moment the cutting of the tooth by the carborundum stone is begun, the assistant floods the stone and the tooth with iced distilled water by means of the device already described. During the entire time of the cutting, the burs and field are alternately flooded with iced water which is removed by the aspirator. Not only is no heat generated by the cutting instruments, but, on the contrary, the temperature of the tooth and surrounding tissues is very materially reduced below normal. A test shows that the water as usually used is delivered to the tooth at a temperature of sixty degrees F. or thereabouts.

### REMOVING THE SEVERED CUSP

As soon as the tooth is severed, the anterior part is usually removed with but little difficulty. The field is then drained by means of the suction machine and examined carefully. Another careful look is taken at the skiagraph before deciding exactly how to proceed to remove the root, the paramount idea being to get it out with as little destruction of the surrounding bone as possible.

### REMOVING THE ROOT

If it is a cone-shaped single root it can probably be released by the *driving of wedges* around its margins between it and the socket, and once it moves, its removal is practically accomplished; but if the root is bulbous, there will probably be trouble ahead.

### CUTTING AWAY THE BONE

When the bone must be cut away from around the root, it can usually best be done with the engine but the occasional use of a chisel is sometimes found desirable.

It must be noted that in order to release the root by means of chisels, a relatively large amount of bone would have to be cut away—the very nature of the instrument renders anything else impossible, while with engine drills, the bone immediately surrounding the remaining parts of the crown and root can be cut away, and the socket thus merely enlarged, if you will, just where it is needed and to any desired degree. Very frequently a very little drilling around the buccal and distal portions of the tooth is all that is necessary.

In other words, with the engine we can cut a little groove in the bone as wide and as deep as we will, while with the chisel we would have to slice off a large portion of the bone.

Upon the completion of this enlargement of the socket, the tooth is frequently started from its seat by the use of these driven wedges or elevators, and once it is well started, the rest is comparatively easy.

The writer is *very partial* to the use of wedges in the removal of root fragments of all kinds. They are driven around the root, where indicated, *between* it and the socket, and frequently roots are thus loosened that could not be removed in any other way with *so little trauma resulting.*

Personally, the writer objects most strenuously to the use of great force by means of an elevator in order to raise such a tooth from its socket. We have all heard of operators who have been so unfortunate as to fracture a jaw under just such circumstances. If we absolutely refrain from using such great force, it should naturally be impossible for such an untoward result to befall us.

So it is that forceps specially designed for the purpose are used when considerable force is needed to start a tooth with unusual roots, which forceps were designed specially to grip the remaining portion of the horizontal tooth after a portion of its crown had been cut off.

### KINGSLEY SCRAPERS

A Kingsley vulcanite scraper has been found to be excellently adapted to use in the mouth. Their wooden handles should be replaced by aluminum. Any machinist can take a piece of one-half inch aluminum rod and turn out a handle to one's own design, and secure the scraper therein.

These make excellent curets, for they cut "both going and coming" and will *curet* as no regular curet will do. And they are very easily kept sharp, which is most important.

### EXAMINING THE FIELD

The removal of the root having finally been accomplished, the soft tissues are next held back so as to expose the margins of the socket, which are kept constantly in view by means of the suction machine while they are examined for splintering or roughness, or possibly a loose or partly loose septum. Splinters there should not be, but nevertheless sometimes they are there and when found must be removed.

### CARE OF THE MARGINS

The margins are then rounded off and made as smooth as possible by means of a carborundum stone in the contra-angle handpiece, as it is most essential that they be left round and smooth, because rough margins or small spicula will surely cause post-operative pain. This is all done under a flow of iced water, of course, and is usually a rather difficult procedure, because of the inaccessibility of the margins. That the soft tissues must be held well back out of the way, and not injured by the stone, is most essential.

We realize that the use of carborundum stones for this purpose will undoubtedly be condemned by some operators, particularly those who may have used them with dissatisfaction to themselves, but we find them far superior to burs for this purpose. Originally, we also objected to the use of stones upon hypothetical grounds, and used surgical burs instead, but finally realizing that we were not getting the margins as smooth as was to be desired by the use of these burs, which evidently were not well adapted to the work, we tried the carborundum stone, and the result was so very much more satisfactory, that we have never used a bur since.

The results obtained by their use prove conclusively that stones are not objectionable—at least when they are used under a flow of iced water. The margins are then gone over wherever possible with a steel scraper, designed for the purpose, and this is also done under iced water. In fact, the parts are drenched with this cold water from start to finish.

### EXAMINING THE MARGINS

After the margins are supposed to be finished, it is most important to run the index finger over them and press the gum firmly over their edges. Sometimes in doing this one will find that the margins instead of being smooth, as was supposed, are so rough that this roughness can be felt through the gum. In that case, the gum is held back again and that roughness smoothed away by means of scrapers or stones, or both, and then it is felt once more as before. This step can not be unduly emphasized.

### CARE OF THE SOFT PARTS

The soft tissues are then replaced in their normal positions. If there are any infected edges, or they overlap, they should be trimmed off.

### CLEANSING THE SOCKET

The socket is then washed out with a couple of ounces of physiologic salt solution by means of the flushing machine, and the space under the loose periostium well flushed out so that no scrapings or débris of any kind can remain.

The socket is washed out from the bottom upward, the only logical method of really clearing it from debris.

If considered necessary, a skiagraph is now taken in order to see if there are any fragments of enamel lying therein, which, of course, should be removed. While the film is being developed, and for ten minutes longer, the socket is slowly irrigated with the iced water. If no skiagraph is taken, the iced water is run through the socket for twenty minutes.

### FINAL TREATMENT OF THE SOCKET

Upon the completion of this cold douche, the contents of the socket are thoroughly aspirated. The wound, probably not bleeding, is made to bleed, and the socket thus filled completely with nice fresh blood. If the soft tissues are opened up for a sufficient length to warrant it, one or two stitches are taken, otherwise not.

If the patient lives in the city, dermal sutures, or others of like character, are used, as the patient can return for their removal when necessary. But if the patient is from the country, then the Lukens' special exodontia sutures are used, which are absorbed and, therefore, require no removal.

### TREATMENT OF THE PATIENT

The patient is now given five grains of pyramidon and put in the rest room for an hour, *no less,* with an ice bag on the cheek. Then full written instructions, for further treatment, should such become necessary, are given and the patient dismissed.

Now if (and this is a great big IF) all these details have been correctly carried out, the patient should report the following morning that no sedatives had been taken after leaving the office; possibly, that he had been to a picture show; that a good night's rest had been had, and breakfast had been eaten as usual. The mouth should open as wide as usual, the operative field should look pink and fine, and *nothing further whatsoever should be done to the case.*

If from the country, the patient can leave that afternoon. Frequently when the operation is performed in the morning the patient leaves for home in the evening, but upon general principles it is preferable for the patient to stay over night just as a matter of prudence.

When this operation is performed in the ideal manner as described, the patient has absolutely no post-operative stage. We do not mean by this that the patient is not aware that something has been done, but we do mean that the patient can go to his office and continue his work for the day or play golf if he chooses, or go shoppnig or to the picture show if she so elects.

In ordinary cases, no pain should follow the operation, though *sometimes* there may be a slight swelling of the face and possibly slight trismus—slight discomfort, that is all. However, sometimes a cog does slip, and then trouble —but, when there is trouble, we *know* a cog did slip, or a "monkey wrench was dropped in the gears"—something went decidedly wrong, and it was probably not the fault of the patient.

### AFTER-PAIN

Should the patient return a *few days after the operation* reporting "deep, burning pain in the socket," the fact can at once be recognized that there is trouble with one of its walls, and the best thing to do is to anesthetize the parts, open up and look for it. If it is a rough margin, smooth it down. If it is a splinter working loose, remove it. The sooner this is done, the better, but it is not always so easy of accomplishment in these inaccessible places.

### CONTRIBUTORY FACTORS

We are thoroughly convinced that the satisfactory results usually obtained in the operation are largely due to two contributory factors—both negative: the one, the elimination of sponging, and the other, the non-packing of sockets.

### SPEED

Never once has it been suggested that this operation is either rapid or easy. Personally we have no ambition to take out *deeply embedded lower third molars* in a few minutes. We would much prefer to spend an hour on any such operation, and thereby eliminate the necessity for further treatment than to spend only ten or twenty minutes upon the work, only to be followed by numerous after-treatments rendered necessary by the speed. While the various steps of this operation call for most accurate and delicate handiwork, they are not beyond the ability of any good and careful operator. The same painstaking attention given to its details that are essential to the accomplishment of any other difficult dental operation is all that is needed.

IDEALS

The ideal result, which we should all strive for, in the removal of impacted teeth should be that the operation itself should not be a strenuous one for the patient, and such a one that it need not be performed in a hospital. The post-operative results should not be more serious than those to be expected after the extraction of an ordinary tooth. This is the ideal result which we should strive for, irrespective of the character of the operation which we select.

For those who achieve these results by the use of mallet and chisel, 'twere folloy to consider the method here presented, but for those who are not accomplishing such results, the writer earnestly urges the careful consideration of the operation now presented.

CONSIDERATION OF THE PATIENT

The consideration of one's patient (1) physically, (2) mentally and (3) financially should be considered of paramount importance.

1. Every means should be employed to lessen the patient's physical discomfort.

2. The patient's mind should be relieved as much as possible, and never anything said to cause unnecessary fear or distress.

3. The patient should never be put to unnecessary expense. If the patient is placed in a hospital for the operation, there will be the usual charge for the room and the fees for the operating room and the anesthetist if ether is used, and other incidentals as well. If the operation is performed in one's own office, there will be no such expenses, which would naturally appeal to every one, but more so to patients in moderate circumstances.

### INSTRUCTIONS TO PATIENTS

I have long since appreciated the fact that giving patients verbal instructions was usually time wasted; therefore, *when necessary* after extraction, I give them a typed copy of the following. It is placed in a self-addressed envelope and returned when of no further use.

### CARE OF THE MOUTH AFTER TOOTH EXTRACTION

*Simple Extractions.* Under ordinary circumstances, after a simple extraction, the mouth requires no more care than it usually receives. Such wounds in the mouth heal rapidly by the cleansing and healing properties of the saliva, though a warm, weak solution of Pond's Extract or Tichenor's Antiseptic used as a wash may prove grateful and beneficial.

Sometimes, however, the unexpected does happen; the socket may not heal rapidly and suffering may ensue. In all such cases, the patient should return at once for examination and to receive whatever attention may be indicated.

*Complicated Extractions.* After severe or complicated operations have been undergone, more especially those in which the tissues were infected at the time of the operation, the patient must expect more or less discomfort or pain, according to the conditions of the case, and should always carefully follow the instructions which have been given for their relief.

### TREATMENT AFTER TOOTH EXTRACTION

*Never* use a hot-water bag or electric pad *directly* on the face for any kind of pain caused by a tooth, or tooth extraction.

If an ice-bag is placed on the face immediately or soon after the extraction of a difficult tooth, it may prevent swelling of the face.

After the face has swollen, *moist heat* should be applied to the face. Antiphlogistine is the best agent for this purpose. This must be applied directly to the face, covered preferably first with a piece of rubber dam, gutta-percha tissue, or even waxed paper, if nothing better can be had, over which a pad of linen (an old handkerchief will answer) should be placed and then a bandage applied to hold it in place. This should be kept in place all night, and, if desired, a hot-water bag or electric pad can be used *over this.*

Hot, weak solutions of Tichenor's Antiseptic or Pond's Extract can be used *in the mouth,* at frequent intervals, to advantage.

In some severe cases, poultices made of powdered slippery elm bark used *in the mouth* will give relief. (Instructions further on.)

Once in a great while a patient will report that hot applications appear to aggravate the pain. In such cases, cold applications, of course, should be used.

In addition to these local applications, five grains of aspirin, to be repeated, if necessary, may give relief in mild cases. Pyramidon in five-grain doses, at intervals of an hour or two, is still more efficacious. Not more than twenty-five grains of pyramidon should be taken within twenty-four hours. In very severe cases where these give no relief, codein should be used, upon prescription only. A laxative should be taken if necessary.

Sometimes the thin margin of a socket is strained or cracked and a small piece will work out later on, and is usually mistaken by the patient for a piece of root. During this process of exfoliation, considerable pain may result, but it can not cause any serious trouble.

Again, during the extraction of a tooth, the tiny end of a crooked root may break off, *and sometimes,* in order to remove this, such extensive cutting of the bone would be necessary, that to allow it to remain might prove to be the lesser evil. As a rule, such a root fragment will do no harm, and later on it will probably come to or near the surface where it can easily be removed with but little difficulty. However, it is *always* better to remove the root fragment if it can be done without undue injury to the surrounding parts.

*Hemorrhage.* Secondary hemorrhage from a tooth socket, while most unpleasant, is not necessarily alarming. It usually can be stopped by making a wad of gauze or a piece of linen cambric, placing this over the socket and biting down upon it, thus stopping the flow of blood by pressure. This pressure should be kept up continuously, *without releasing,* for a good many minutes—thirty or longer, if possible—by which time a good blood clot should have formed. In obstinate cases, the dentist or physician must use more efficient means.

*Allaying Pain.* Heat applied to the seat of pain usually proves to be the best remedy. Within the mouth it can best be applied by means of poultices used as follows:

Make three small bags, out of old linen cambric, about the size of the end of your thumb, and about one and one-half inches long. Fill these half full of powdered slippery-elm bark and sew them up. Place these in a saucepan of hot water and keep the water hot while in use. Place one of these poultices in the mouth over the seat of pain—usually between the cheek ond the socket or tooth—and hold it there until cool. Then place it back in the hot water and put a hot one in the mouth. Keep this up for one hour by the clock, changing the poultices just as fast as they get cool.

Repeat this performance two or three times a day, as long as necessary.

Rubbing the face with camphorated vaseline or oil for ten or fifteen minutes at a time is soothing and may hasten the reduction of swelling.

If one would just stop a moment and consider what it means to forcibly tear out a tooth which is set firmly in the jaw, the wonder is *not* that there is *some* suffering afterward, but that the consequences are not always really very serious. The fact is that the removal of *some teeth* is a surgical operation of no mean degree.—*Cosmos,* for February, 1921.

